# Association of Physical Activity with the Prevention and Treatment of Depression During the Postpartum Period: A Narrative Review

**DOI:** 10.7759/cureus.44453

**Published:** 2023-08-31

**Authors:** Monique N. Fotso, Natalie A Gonzalez, Raghavendra R Sanivarapu, Usama Osman, Abishek Latha Kumar, Aishwarya Sadagopan, Anas Mahmoud, Maha Begg, Mawada Tarhuni, Safeera Khan

**Affiliations:** 1 Obstetrics and Gynecology, California Institute of Behavioral Neurosciences & Psychology, Fairfield, USA; 2 Pediatrics, California Institute of Behavioral Neurosciences & Psychology, Fairfield, USA; 3 Pulmonary and Critical Care Medicine, California Institute of Behavioral Neurosciences & Psychology, Fairfield, USA; 4 Internal Medicine, California Institute of Behavioral Neurosciences & Psychology, Fairfield, USA

**Keywords:** edinburg postnatal depression score (epds), postnatal depression, physical activity, exercise, postpartum depression

## Abstract

The benefits of physical activity during pregnancy or postpartum are auspicious, especially in preventing and treating postpartum depression. This review aimed to examine the relationship between exercise and postpartum depression in terms of prevention and treatment. The goals were to determine if exercise alone is sufficient for this purpose and to attract attention to the kind, intensity, and duration needed to achieve this purpose. A literature review was conducted in PubMed, PubMed Central, MEDLINE, and Cochrane libraries. The search terms were “physical activity," "postpartum depression,” "postnatal depression," and "exercise.” Physical activity can be a preventative measure and a treatment aid for pregnant or postpartum women with depressive disorders. However, the exercise protocol should include a support/wellness program to achieve better and more remarkable results.

## Introduction and background

"I felt like a zombie. I couldn't access my heart. I couldn't access my emotions. I couldn't connect. It was terrible…The hardest part for me was acknowledging the problem. I thought postpartum depression meant you were sobbing every single day and incapable of looking after a child. But there are different shades of it and depths of it, which is why I think it's so important for women to talk about. It was a trying time. I felt like a failure." Gwyneth Paltrow

A female goes through enormous changes all through pregnancy and labor, as well as critical physical and emotional changes, to such an extent that her entire world is impacted, particularly her familial and interpersonal milieu [1,2]. Postpartum depression (PPD), defined as any depressive disorder that occurs from the onset of pregnancy or within four weeks of delivery, is one of the most common complications of childbirth [3]. Around 6.5% to 20% of women are affected with PPD, with more risk in the first 12 months after birth [1,4-6]. According to a recent study by the Centers for Disease Control and Prevention (CDC), approximately one out of every eight women suffer from symptoms of PPD [7]. After giving birth, many women suffer from emotional disturbances, such as sobbing, bewilderment, mood swings, anxiety, and depression. These symptoms often start within the first postpartum week and persist for several hours to a few days. However, if they persist for more than two weeks and significantly impair the mother's ability to function, it is PPD [1-4,6,8].

It is crucial to identify pregnant and postpartum women with depression or depressive symptoms because, if left untreated, it could have devastating effects on both the mother and child and, most importantly, on the mother-infant relationship [4,8-12]. For this reason, the American College of Obstetricians and Gynecologists (ACOG) recommends the screening of patients by obstetricians-gynecologists, as well as other obstetric care providers at least once during the perinatal period for depression and completing a full assessment of mood and emotional well-being during the comprehensive postpartum visit for each patient [13]. The ultimate objective in the postnatal period is to maintain excellent well-being, but if depression endangers the patient's well-being, the focus turns to assuaging the severity of the depression. It is possible to reduce this severity using non-pharmacological strategies such as psychotherapy and exercise or pharmacological strategies such as antidepressants or hormonal therapy [5,14]. In a research by Blumenthal et al., exercise improved depressive symptoms in the general population and was almost on par with pharmacotherapy and psychotherapy [15]. A meta-analysis conducted by Poyatos et al. revealed that physical activity during pregnancy and postpartum significantly reduced postpartum depressive symptoms [3].

Participating in physical activity during or after pregnancy can provide numerous advantages. Engaging in physical activity while pregnant can lower the chances of developing gestational Diabetes, operative vaginal delivery, or cesarean birth. It also decreases postpartum recovery time and the risk of PPD [16]. The benefits of postpartum exercises include reducing fatigue and promoting better sleep, strengthening and toning abdominal muscles, weight loss and return to prepregnancy weight, prevention of lactation-associated bone loss, and improving fitness level. Exercise may also give new mothers a sense of control and accomplishment, which can improve mood, self-confidence, and self-esteem [16-19]. For these reasons, the recommendation from ACOG on physical activity is a minimum of 150 minutes of moderate-intensity exercises per week for pregnant and postpartum women, with all necessary modifications, pieces of advice, and clearances from obstetricians taken into account [16]. This review aims to demonstrate the value of exercise in avoiding PPD. The goals were to determine if exercise alone is adequate for this purpose and to attract attention to the kind, intensity, and duration of exercise to minimize the risk of developing or aid in treating depressive disorders during the postpartum period.

## Review

Methods

In this narrative review, four databases were methodically searched - PubMed, PubMed Central (PMC), Medical Literature Analysis and Retrieval System Online (MEDLINE), and Cochrane Library [20-22] - to find articles on the topic using Medical Subject Headings (Mesh) suitable keywords such as "physical activity," "postpartum depression," "postnatal depression," and "exercise." The final combined Mesh Strategy for PubMed, PubMed Central, and Medline was "Depression, Postpartum" [Majr] AND "Exercise Therapy" [Mesh]. The search of the Cochrane library using the final MeSH strategy as MeSH descriptor: [Depression, Postpartum] explode all trees, MeSH descriptor: [Exercise] this term only using Boolean AND was also fruitful.

Inclusion and exclusion criteria

Studies were only selected if (1) women included in the study were pregnant or had just given birth; (2) these women were established to be depressed or at risk of depression in the postnatal period; (3) physical activity alongside usual care was assessed during pregnancy into the postnatal period or began during the postnatal period in comparison to just usual care; (4) outcome assessed was a decrease in depressive symptoms measured by a depression scale; and (5) publication was within the last 15 years (2007-2022). Articles were rejected if (1) they were published outside the 15-year considerable period; (2) physical activity was done in the non-pregnant and non-postpartum population; (3) were non-English publications or grey literature; and (4) there was no free access to the full version of the article.

Screening

**Figure 1:**
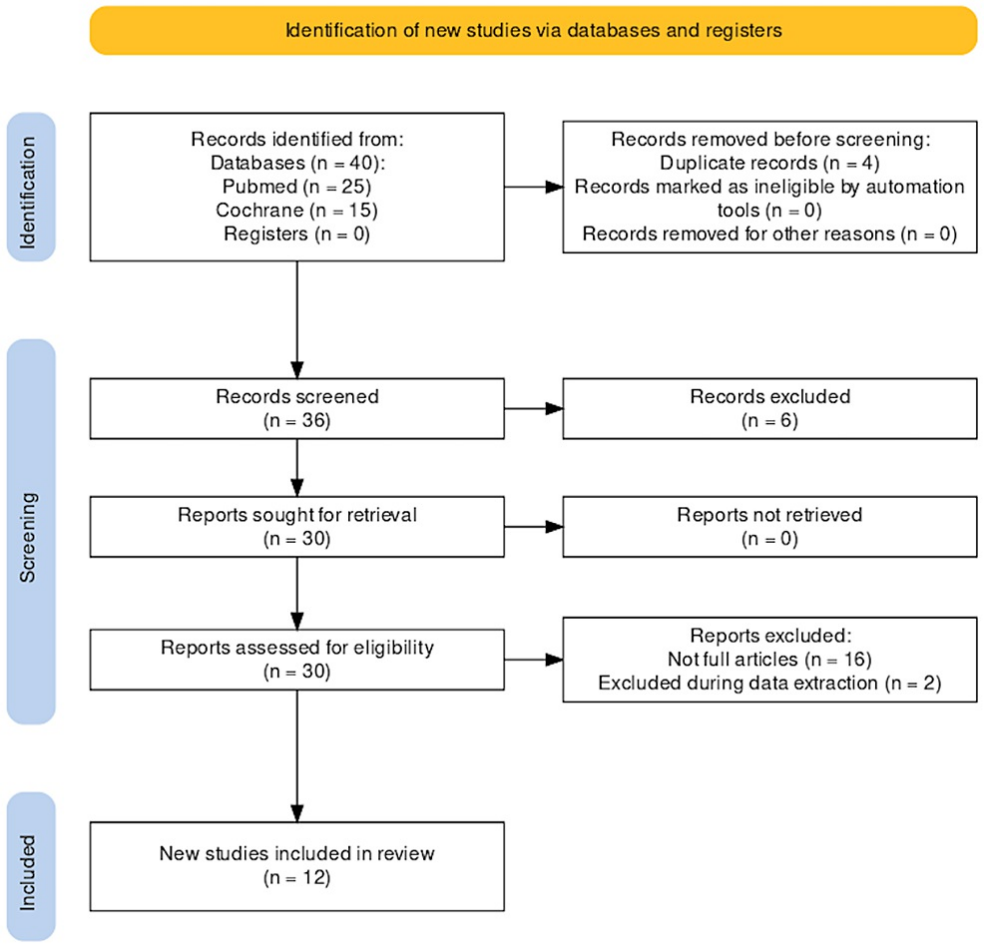
PRISMA 2020 flow diagram PRISMA, Preferred Reporting Items for Systematic Reviews and Meta-Analyses

A total of 40 articles were found, of which 10 were rejected based on the analysis of duplicates, titles, and abstracts. Sixteen articles were further excluded after screening for the availability of full text. A total of 14 articles were read, and, finally, 12 studies were included in the review, as depicted by the Preferred Reporting Items for Systematic Reviews and Meta-Analyses (PRISMA) 2020 flow diagram in Figure 1 [23].

Results

This review included nine randomized controlled trials, one qualitative study, one quasi-experimental study, and one meta-analysis. All the studies used the Edinburg Postnatal Depression Score (EPDS) to assess depressive symptoms [24]. These studies were conducted in several countries: Australia [25-27], Turkey [14], Brazil [28], Spain [3], United Kingdom [29], Norway [30], Taiwan [31,32], Iran [33], and the United States [34]. Physical activities that were undertaken in the intervention groups included the following: pram walking [27,29,31], gentle stretching [32], aerobic and strengthening [30], aerobic activities and pregnancy-specific floor exercises [28], stretching and breathing exercises [33], and "Mother and Baby" (M&B) program including specialized exercises (cardiovascular and strength exercises) [26]. Three studies did not specify the kind or type of exercise the intervention group undertook [14,25,34]. The women who were part of the control group were given standard care. The exercise programs for the intervention groups in most of the studies were undertaken in the postpartum period except for three studies: two studies in which the exercise program was conducted during pregnancy and one study where it began during pregnancy and continued into the postpartum period. The primary features of the studies are shown in Table 1.

**Table 1:** Study characteristics EPDS, Edinburgh Postnatal Depression Scale; BDI, Beck Depression Inventory; PABS, Positive Affect Balance Scale; PSQS, Postpartum Sleep Quality Scale; PFS, Postpartum Fatigue Scale; FIF, Fatigue Identification Form; SCID-I, Structured Clinical Interview for DSM-IV Axis I Disorders; SSI, Social Support Interview; NA, not available; h/w, hours per week

Author (year), country	Sample size (n)	Research tool	Type of exercise intervention	Length (weeks)	Intensities	Frequency (sessions per week)	Main result
Poyatos-Leon et al. (2017), Spain [3]	932	EPDS, BDI (Chinese version)	Stretching and breathing exercises, a walking program, cardiovascular exercises, mixed cardiovascular and strength exercises, pilates and yoga exercises, and home-based programs	NA	Low, moderate, or moderate to high	Varied from 1 to 5 days per week	Physical activity during pregnancy and the postpartum period is associated with a lower incidence of postpartum depressive symptoms.
Özkan et al. (2020), Turkey [14]	80	EPDS	NA	4	Mild-to-medium and medium-to-severe levels	5	There was a statistically significant difference in the post-test EPDS mean scores, but none was found in the pretest scores between the exercise and control groups.
Apostolopoulos et al. (2021), Australia [25]	20	EPDS	Semi-structured telephone interviews	NA	NA	20 minutes	Postpartum women with depressive symptoms reported that their physical and social environments influenced their physical activity and screen time. However, only physical environment was found to influence screen time.
Norman et al. (2010), Australia [26]	130	PABS, EPDS	“Mother and Baby” (M&B) program including specialized exercise	8	Moderate	1	There was a significant improvement in well-being scores and depressive symptoms of the intervention group compared to the control group.
Armstrong et al. (2004), Australia [27]	19	EPDS, SSI	Pram walking	12	Moderate	3	Overall improvement in depressive symptoms and fitness levels in the intervention group compared to the control group at the 12-week mark.
Coll et al. (2019), Brazil [28]	639	EPDS	Aerobic, strength training, and pregnancy-specific floor exercises	16	Moderate	3	No significant differences were observed between the two groups in the primary analysis.
Daley et al. (2008), United Kingdom [29]	38	EPDS	Pram walking	12	Moderate	5	No significant difference in EPDS scores was found between the groups
Songoygard et al. (2012), Norway [30]	855	EPDS	Aerobic and strengthening exercises, endurance training	12	Moderate to high	Minimum 3	There was no significant difference in postnatal depression between both groups. However, a subgroup of women in the intervention group who did not exercise before pregnancy had a reduced risk of developing postpartum depression compared to the control group.
Liu et al. (2021), Taiwan [31]	104	PSQS, EPDS, PFS	Pram walking	12	NA	3	There was no significant difference between both groups regarding either fatigue or depression at four weeks and 12-week post-tests, but a significant improvement with sleep inefficiency symptoms was observed.
Heh et al. (2008), Taiwan [32]	80	EPDS	One h/w at the hospital and two sessions at home	12	NA	3	There was a decrease in EPDS scores at five months postpartum compared to the control group
Mohammadi et al. (2015), Iran [33]	127	EPDS, FIF	Home-based stretching and breathing exercises	12-18	Low intensity	3	There was no significant difference in EPDS and FIF scores between the three groups after one- and two-month post-test.
Lewis et al. (2021), USA [34]	450	SCID-I, EPDS	11 telephone sessions	26	Moderate or vigorous intensities	5	Low rates of depression based on the SCID scores at both six and nine months. Significant difference in EPDS at six months in the wellness intervention group compared to the usual care group.

Discussion

PPD is a prevalent issue after childbirth, and despite its harmful effects, it often goes undiagnosed and untreated [3,5]. One of the main reasons for not treating PPD is the issue of mental illness stigma. Other reasons include the effects of pharmacological treatment on breastfeeding, access to care, and personal beliefs [9]. Exercise acts as a natural antidepressant, which is why it has been studied as an alternative or adjunct to treatments for depression [35]. Exercise increases the expression of neurotrophic factors such as brain-derived neurotrophic factor (BDNF), which is typically low in depressed people. This factor is responsible for neuroprotection, neurogenesis, and synaptic plasticity. Exercise also increases the availability of serotonin and noradrenaline, regulates the hypothalamic-pituitary-adrenal (HPA)-axis activity, and reduces the production of inflammatory cytokines. In addition, exercise stimulates the production of growth hormone and insulin-like growth factor, which regulates sleep, cognitive function, and mood [5,35,36].

*Physical Activity During Pregnancy* 

Comparing the results from the evaluation of the articles included in the review suggests a plausible benefit of exercise during the puerperium period in decreasing the symptomatology of PPD. The efficacy of regular exercise during pregnancy on depressive disorders in pregnancy and the postpartum period was examined by Coll et al. [28] over four months. Women from the intervention group followed a structured, supervised, moderate-intensity exercise protocol for 60 minutes thrice a week. This intervention started between the 16th and 20th week of pregnancy till the end of the third trimester (32-36 weeks of gestation). The primary analysis did not observe significant differences between the intervention and control groups. It was stipulated that the failure to observe a significant difference in the primary analysis may have been due to the low compliance to exercise in the intervention group. However, the mean antenatal EPDS scores were significantly lower among women in the intervention group compared to the control group when the compliance rates were high, though it was not sustained into the postpartum period. Mohammadi et al. [33] also assessed the influence of exercise during pregnancy and the postpartum period on PPD. The participants were divided into three groups: the control group, a group with exercise intervention during pregnancy, and a group with exercise intervention during pregnancy and the postpartum period until two months after delivery. The interventions were low-intensity stretching and breathing exercises, which had to be done thrice a week, 20-30 minutes each, until delivery for the group during pregnancy. The second intervention group participants were instructed to adjust the frequency and duration of the exercises during the first postnatal month but to return to the same frequency and duration as the first intervention group during the second postnatal month. The EPDS and Fatigue Identification Form (FIF) scores were obtained at baseline, one month postpartum, and two months postpartum for the depression and fatigue scores, respectively. The results showed an over-reduction in the EPDS and the FIF at one and two months postpartum. However, the mean rank difference between the intervention and control groups was not statistically significant. Again, this result resulted from the low compliance with the exercise program and the low-intensity exercise done by the intervention group.

Similarly, Songoygard et al. [30] conducted a study in which pregnant women between 20 and 36 weeks’ gestation participated in a supervised training (60 minutes, once a week for 12 weeks) and a 45-minute self-training exercise program at home. Three months post-delivery, the participants completed the EPDS, which did not show a significant difference in the level of depression between the intervention group and the control group during pregnancy. However, the study's researchers observed a lower rate of depression among women in the intervention group who were physically inactive before the study compared to those in the control group. Finally, Poyato-Leon et al. [3], after analyzing 12 articles in their meta-analysis looking at the effects of exercise-based interventions on PPD, concluded that the lower incidence of postpartum depressive symptoms in the participants of the intervention groups was linked to physical activity during pregnancy and the postpartum period compared to the participants in the control group.

Though the compliance rate to exercise was low in most of these studies, some reduction in the post-delivery EPDS was observed in the intervention groups compared to the control groups.

 *Physical Activity During the Postpartum Period *

Daley et al. [29] investigated the practicability of exercise as an intervention for PPD. This study was conducted over 12 weeks, where the participants in the intervention group were offered two one-to-one exercise consultations and encouraged to participate in moderate-intensity activities for at least 30 minutes per day, five times a week. EPDS scores were recorded at baseline and the 12-week mark. Although no significant difference was found between the groups regarding EPDS scores, the exercise group reported a high self-efficacy for exercise scores, which was the primary outcome. Liu et al. [31] also found no significant difference in the EPDS scores when they examined the outcomes of walking exercise in postpartum women with disordered sleep. Women in their sixth postpartum week were recruited to perform long-step pram walking for 20-30 minutes per session thrice a week for three months. The outcomes were measured at four weeks and the end of the intervention. The study's primary outcome was sleep quality, which significantly improved. Conversely, fatigue and depression, secondary outcomes, had no difference in scores of the Postpartum Fatigue Scale (PFS) and EPDS, respectively, between the groups.

However, Armstrong and Edwards [27] showed a direct association between improved fitness levels and improved depressive symptomatology of the participants in the intervention group compared to the social support group when they investigated the effects of pram walking in reducing depressive symptoms. In addition, Ozkan et al. [14] demonstrated that in postpartum women at risk of PPD after exercising for four weeks at different phases of intensities (mild and medium exercises at least 30 minutes a day, five days a week for two weeks and medium and severe for the last two weeks), the depressive symptoms of the participants in the intervention group were significantly decreased compared to the control group. Heh et al. [32] also found that primiparas who exercised (45-minute, whole body, gentle stretching exercise program plus warm-up and cool-down exercises) an hour thrice a week for three months had decreased EPDS scores and improved physical fitness levels and overall psychological well-being at five months postpartum compared to the control who received just standard care. Additionally, the intervention group participants received a support program that consisted of three sessions a week for three months. This result is supported by earlier research by Norman et al. [26], in which they evaluated the effects of exercise and healthcare education programs on the psychological well-being of new mothers. They found out that one-hour group exercise, which involved cardiovascular and strength components, and 30-minute education sessions for eight weeks had a tremendous impact on the well-being scores and depressive symptoms of the participants in the intervention group compared to the control group.

Moreover, Lewis et al. [34] examined the influences of exercise and wellness interventions on preventing PPD and perceived stress. They randomized postpartum women who had a history of depression and exercised less than 60 minutes per week into three groups: the standard care group, the telephone-based exercise intervention group, and the telephone-based wellness/support intervention group. The wellness/support intervention was included to target health behaviors such as eating and sleeping habits to isolate the effects of exercise alone on PPD and perceived stress. The intervention was any moderate-to-vigorous exercise intensity program done for 30 minutes per session five days a week. Structured Clinical Interview for DSM-IV Axis I Disorders (SCID-I) and EPDS scores were obtained at baseline, six months, and nine months post-randomization. They found that a combination of wellness interventions and exercise influenced PPD, as shown by lower rates of SCID-I and EPDS scores six months post-randomization in the wellness/support intervention group compared to the standard care group. However, the individuals in the exercise intervention group did not have lower levels of depression than those in the usual care group.

Based on the research cited above, it was found that engaging in physical exercise not only helped alleviate the depressive symptoms experienced by those in the intervention groups but also led to improvements in exercise tolerance, fitness levels, and sleep quality. Additionally, it had a positive impact on the psychological well-being of the participants.

Exercise and Wellness/Support Programs

Various exercises were done in the studies under review and with different intensities (low, moderate, and vigorous), with the most common being moderate-intensity exercises. Regarding duration, the first noticeable change in the depressive symptoms or EPDS scores was after a minimum period of four weeks. Nevertheless, we also noted that of all the studies in the review, the most successful ones integrated some form of support or wellness intervention alongside the exercise intervention.

Apostolopoulos et al. [25] postulated that social support groups of postpartum women experiencing similar situations were crucial in not only increasing adherence or compliance to the exercise programs, which further accentuates the benefits of exercise on PPD, but also in providing the necessary support needed by these women in day-to-day activities, and emotional and mental states. Additionally, the participants' solution toward realizing the goal of compliance with the exercise regimen was awareness-raising, education, and guidance on resuming physical activity post-childbirth. Women in the study conducted by Heh et al. [32] mentioned that the exercise support program benefited them because it helped them understand themselves better emotionally and learn about more positive ways of coping with PPD. Thus, as stated by Lewis et al., "...a wellness intervention in addition to exercise is needed to impact depression" [34].

Limitations

This study had its weakness in that only free full-version articles were reviewed; thus, not all types of exercises were studied. We believe having a wide variety of exercises would have given a better picture of the benefits of physical activity on PPD. However, nonetheless, the study's results should still be considered.

## Conclusions

After reviewing the findings, it can be concluded that physical activity plays a significant role in decreasing depressive symptoms in the postpartum period by acting as a natural antidepressant. Still, to reap the benefits of exercise as a therapeutic approach to PPD, it should be of moderate-to-vigorous intensity of at least 150 minutes per week as recommended by ACOG. Nevertheless, for exercise to have a good impact on the physical well-being and, most significantly, the mother’s mental well-being, it should go hand in hand with a wellness or support program that targets the behavioral components of a healthy lifestyle, such as eating, sleeping, and stress reduction. Also, mothers should be educated and suitably guided on when, how, and what to do about resuming physical activity after childbirth. It is highly recommended that healthcare professionals promote physical activity for women during and after pregnancy. To further combat PPD, it is important to conduct research that will identify and implement intervention strategies that will target the health behaviors and encourage adherence to exercise during pregnancy, especially in the postpartum period.
